# High-Quality Video Watermarking Based on Deep Neural Networks for Video with HEVC Compression †

**DOI:** 10.3390/s22197552

**Published:** 2022-10-05

**Authors:** Maciej Kaczyński, Zbigniew Piotrowski, Dymitr Pietrow

**Affiliations:** Faculty of Electronics, Military University of Technology, 00-908 Warsaw, Poland

**Keywords:** neural network, watermark, deep learning, property verification, copyright protection, video, HEVC, H.265, YUV420, YUV420p

## Abstract

This article presents a method for transparent watermarking of high-capacity watermarked video under H.265/HEVC (High-Efficiency Video Coding) compression conditions while maintaining high-quality encoded image. The aim of this paper is to present a method for watermark embedding using neural networks under conditions of subjecting video to lossy compression of the HEVC codec using the YUV420p color model chrominance channel for watermarking. This paper presents a method for training a deep neural network to embed a watermark when a compression channel is present. The discussed method is characterized by high accuracy of the video with an embedded watermark compared to the original. The PSNR (peak signal-to-noise ratio) values obtained are over 44 dB. The watermark capacity is 96 bits for an image with a resolution of 128 × 128. The method enables the complete recovery of a watermark from a single video frame compressed by the HEVC codec within the range of compression values defined by the CRF (constant rate factor) up to 22.

## 1. Introduction

Nowadays, the issue of securing ownership rights to materials in digital form is one that requires further development. There are various ways of securing digital materials, such as DRM (Digital Rights Management), to protect against copying or illegal processing. A popular method of securing the copyright of video materials is to embed a watermark in them. The embedded watermark may or may not be visible. In the first case, a visible watermark is added to the video, often in the form of the logo of the TV station. The visible watermark has a high degree of ease of implementation and recognition, but it also obscures a portion of the video, altering the viewer’s visual experience [1,2]. In the case of transparent watermark embedding, no additional graphic element is introduced in the form of a visible watermark and, what is more, the person reproducing the material in question may not know that it is protected in this way due to its invisibility [3,4]. Watermarking methods have also evolved with the development of their detection and removal [5,6,7,8]. These methods address the detection and removal of visible as well as transparent watermarks.

The implementation of embedding a visible watermark involves permanently adding a visible logo to the video, identifying the owner of the copyright to the video in question. On the other hand, the implementation of a transparent watermark is a more complex issue requiring consideration of fundamental issues related to the subject, such as capacity, visibility, resistance to changes of the carrier, and reversibility of changes made to the image.

This publication will present a system for protecting ownership of video materials based on embedding a transparent watermark in the video. In the case of transparent image watermarking, there are descriptions in the literature of more elementary methods, such as the use of the least significant bit [9], based on frequency analysis, such as the wavelet transform [10], as well as more complex methods [11] that are a specific combination of basic methods or even dedicated to particular video codecs and use their characteristics for their operation. With the popularization of artificial neural networks (ANNs), watermark embedding methods using them have also emerged [12,13,14,15,16,17].

Classical methods, as well as those based on ANNs, have strengths and weaknesses due to the characteristics of their operation. Both classical and ANNs-based methods have the potential for further development to improve the performance of existing methods or adapt them to new issues, such as new video codecs. The method presented in this article is based on the use of deep neural networks (DNNs) because with the right choice of DNN architecture and proper design of the training set, trained DNN should have the ability to find properties of the problem being analyzed that may not be included or predicted in a manually written algorithm.

The aim of this article is to present an improved method compared to the previously published method for embedding a watermark in a high-quality video based on DNN under High-Efficiency Video Coding (HEVC) [18,19,20,21] codec compression conditions [22]. The main research problem was to ensure the resistance of the watermark under higher HEVC compression levels than in a previous paper [22] and in comparison to other methods. Additionally higher capacity size for the watermark was achieved while providing higher values of carrier PSNR. The presented method of embedding the watermark takes into account the subsampling of the YUV420p chrominance in a way that ensures its recovery while providing high accuracy of the video with the embedded watermark in relation to the original video. The method is based on the use of a DNN autoencoder (using a real HEVC compression channel and mimicked by a trained coder in the training process) and on the use of an adjustable subsquares properties algorithm (ASPA) [22] method for watermark generation in chrominance channels.

The structure of this manuscript is as follows: A discussion of the literature is presented after the introduction. Next, the proposed method will be presented and discussed, taking into consideration the training of the encoder together with the decoder and the training of the coder mimicking the HEVC compression channel. After the presentation of the proposed method, the results of the method will be presented with a discussion. At the end of the manuscript, there are conclusions summarizing the outcome of the study.

## 2. Literature Review

In reviewing the literature on watermark embedding, a distinction was made between classic methods and methods using ANNs.

Classic methods can include methods, such as those based on the least significant bit [23,24,25] or methods based on frequency domain manipulation (discrete wavelet transform, discrete Fourier transform, discrete sine, and cosine transform) [10,26,27,28,29,30,31]. There are many modifications of these methods, such as a combination of the least significant bit method and those based on manipulation of the frequency domain, which includes publications concerning hybrid domains [23,32,33,34].

When discussing the embedding of a watermark in an image, a distinction should be made between embedding a watermark in a static image [23,34,35,36,37] and in a video [14,15,16,17]. The methods used for static images find their application and development in methods for video purposes. An elementary example of an information hiding method is the modification of the least significant bit, which is effective in its simplicity but sensitive to any kind of operation performed on a dynamic image, such as a video image. More resistant are methods based on frequency domain manipulation, where the watermark is embedded in such a frequency band of the image as to produce a watermarked image with the least degradation in quality compared to the original. Images are modified as a whole or in certain areas of interest, which may be parts of the image where it is easier to hide information or, due to the operation of the method for which no overall modification of the image is necessary to hide the information.

Embedding a watermark in a video is a more complex issue than that of a static image. The main problem is to keep the watermark highly resistant while maintaining good video quality. The difficulty itself stems from the fact that the video image, when encoded into a particular format (depending on the DVB-T receiver, Internet TV), is usually compressed to reduce its size several times. This compression is usually multifaceted [38,39] and is carried out not only in the binary symbol domain but is also achieved by downsampling of channels in a given color model, introducing changes in the frequency domain and introducing a motion vector, where such a cascade of source image translations ultimately hinders the possibility of watermark reconstruction.

Multifaceted variations in the source image make it difficult to manually select the appropriate method for watermarking a transparent video image. This has led researchers towards using adaptive methods, such as DNN, for this purpose. Incorporating DNN into the issues of stenography and video watermarking allow a high probability of recovering the watermark and keeping the quality of the watermarked image in high quality [38,39,40] by being able to select optimal image composition and decomposition methods as a result of the training process [41,42,43,44,45,46,47].

Depending on the approach adopted, the watermark can be recovered from a single video frame or from a specific number of frames.

For video watermarking, both classic and ANN-based methods are used [14,15,16,17,48,49,50,51]. Among these are methods dedicated to specific video codecs, including the H.265/HEVC (High-Efficiency Video Coding) codec for which the method presented in this article has been developed [16,17,50,51].

A noteworthy issue is the description of the coefficients describing the degree of accuracy of the watermarked video to the original [52]. The PSNR (peak signal-to-noise ratio) and MSE (mean-squared-error) are popular coefficients and used in this publication. There are also other coefficients that address the determination of the degree of accuracy between images, such as: NMSE (normalized-mean-squared-error), RMSE (root-mean-square-error), or SSIM (structural-similarity-index-measure). At this point, it is important to emphasize that, although the above-mentioned coefficients work well in a general comparison of quality, they are not a definitive guarantee of its quality. It is possible that there will be a small but clearly visible graphic artifact in a large image that does not significantly affect the overall image accuracy value determined mathematically.

## 3. Proposed Method

### 3.1. Presentation of the Concept of the Proposed Method

**Figure 1:**
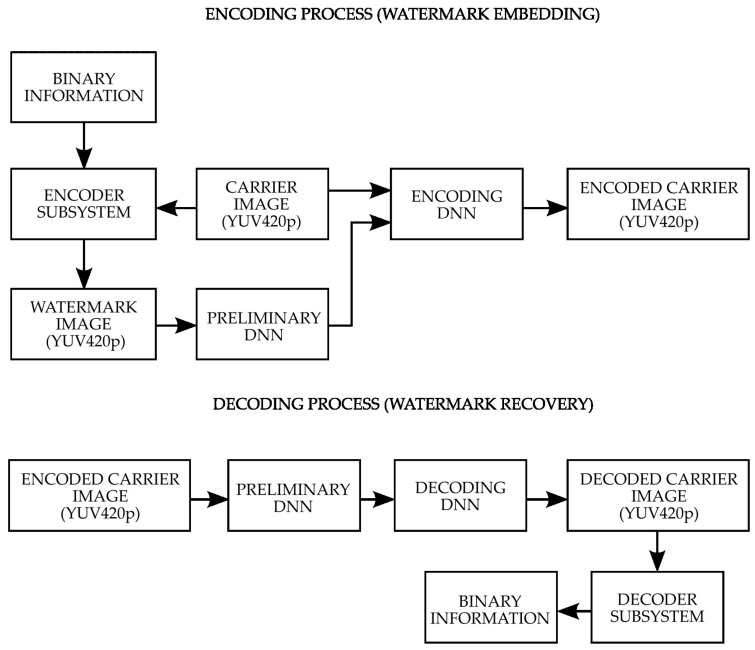
Conceptual diagram of the system.

The presented method is a continuation of the method presented in a previous publication aimed at embedding a watermark in the compression conditions of the HEVC codec using DNN and ASPA (Adjustable Subsquares Properties Algorithm). In the watermarking system concept presented further in this article (Figure 1), the additional factor of YUV420p chrominance subsampling used in video codecs as one of the compression stages allowing an additional reduction in memory requirements at the expense of degrading the quality of the chrominance channels is included.

The system consists of two main components of a watermarking encoder and a watermark reconstruction decoder. Embedding a watermark in an image consists of two stages. In the first stage, the binary character string is transformed via the ASPA algorithm into an image that is optimal for encoding in chrominance channels. In the next step, the watermarked image thus obtained is fed to the DNN encoder input together with the image that will carry the watermark. At the encoder output, an image of the carrier with a transparently embedded watermark is obtained. From the image thus obtained, the watermark can be recovered by feeding it to the input of the decoder and obtaining a reconstructed watermark at its output.

The encoder and decoder subsystems use ASPA for efficient encoding of information in the image, which was discussed in detail in a previous publication [22], however the main idea of the algorithm will be briefly explained here as well.

The area of the square with dimensions n × n is divided in such a way as to form a fixed number of subsquares with dimensions m × m that fit into the square in such a way as to fill all its available space of n × n. Translating this for the case used in this article, the square of 128 × 128 was divided into 16 subsquares of 32 × 32. Each subsquare for a hidden watermark copies the original luminance value (Y-channel) to increase the similarity of the encoded image with the original image and has chrominance values (U and V channels), which are the actual carrier of the hidden information and represent digits of the selected number system, which is the octal in this case. Thus, U and V may take values in the range of digits ⟨0;7⟩. The value in the V channel should be interpreted as a less significant bit and U channel represents the most significant bit.

Each digit is assigned a fixed value during the watermark embedding process. For the decoding process, a fixed range of values is assigned to each of the interpreted digits. The ranges of values used in this method for the decoding process are shown in Table 1.

Two numbers in the octal number system contain 64 combinations of values. This property was used to assign an appropriate ASCII character to a given value. In this way, the letters of the alphabet, as well as numbers and some special characters, were assigned. Characters representing watermark are read from the text file and converted into the appropriate assigned value in the range ⟨0;64⟩ for the encoding needs. Thus, each subsquare in ASPA represents a single character.

The sizes of the main square, as well as the subsquares used, have remained unchanged and are 128 × 128 and 32 × 32, respectively. The numeral system used was changed from senary to octal, increasing the capacity of the watermark from 80 bits to 96 bits. The ANNs used in the article perform calculations over a normalized range of values ⟨0;1⟩ and the encoder and decoder subsystems operate on such values. Table 1 shows the ranges of values for which the individual digits of the octal numeral system are interpreted along with their values when converted to the values of the standard RGB color model.

A watermark is encoded in the U and V chrominance channels, where the digits that correspond to the encoded value are encoded, while the Y luminance channel is copied from the original image carrying the watermark.

**Figure 2:**
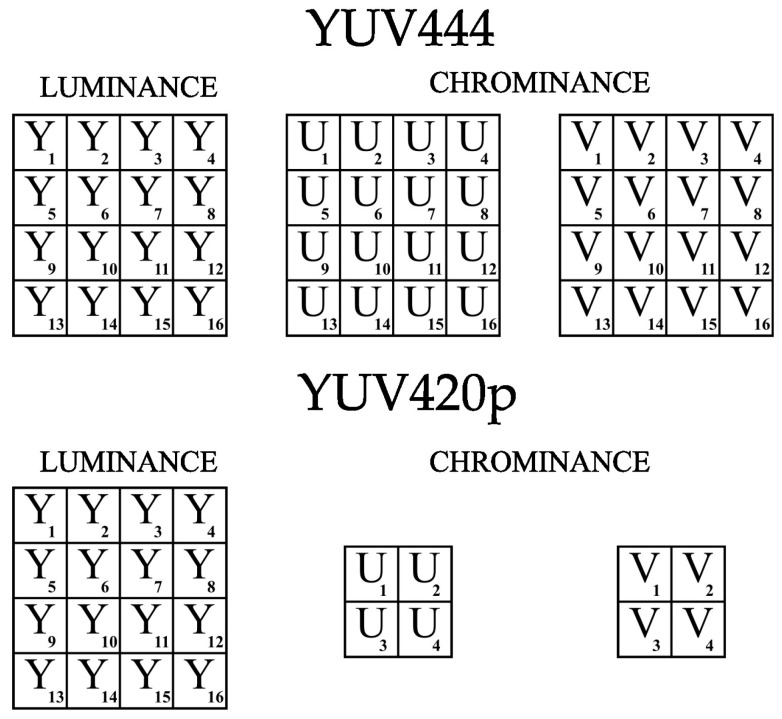
Demonstration of YUV color model for the YUV444 and YUV420p variants.

Despite the use of YUV420p chrominance subsampling for video purposes, the coding algorithm used works well because it features a flexible selection of the size of the subsquares in which the bit strings are placed. Figure 2 shows a graphical comparison of YUV color model for the YUV444 and YUV420p variants.

### 3.2. HEVC Compression Learning Process

In order to increase the accuracy of the embedded watermarked image compared to the original image and to increase the tolerance range of HEVC compression, an additional training element was introduced in the training process of the encoder and decoder that mimics HEVC compression, hereafter referred to as the DNN coder. In this subsection, the training process for the aforementioned element will be presented, while the training of the encoder embedding the watermark, as well as the decoder performing the watermark reconstruction, will be presented in the next subsection. In order to carry out the training process of the DNN coder mimicking HEVC compression, the following objective function relationships were used:

(1) $$MSE_{OrgCodImg}=\mathrm{MSE}\left({\mathrm{Image}}_{Original},\;{\mathrm{Image}}_{Coded}\right)$$

(2) $$MSE_{HevcCodImg}=\mathrm{MSE}\left({\mathrm{Image}}_{Hevc}\left(CRF\right),\;{\mathrm{Image}}_{Coded}\right)$$

(3) $$\mathrm{EpochChange}\left(i\right)=i\cdot 0.001$$

(4) $$L_{CodLoss}\left(i\right)=\left(\left(\left(1.01-\mathrm{EpochChange}\left(i\right)\right)\cdot MSE_{OrgCodImg}\right)\;\;+\left(\left(0.50+\mathrm{EpochChange}\left(i\right)\right)\cdot MSE_{HevcCodImg}\right)\right)\;i\;\epsilon \left(0;300\right\rangle$$

(5) $$\nabla L_{Cod}\left(i\right)=\frac{\partial L_{CodLoss}\left(i\right)}{\partial Var_{Cod}\left(i\right)}$$

where $\mathrm{MSE}\left({\mathrm{Image}}_{1},{\mathrm{Image}}_{2}\right)=\frac{1}{N_{j}}\overset{N_{j}}{\underset{i=0}{\sum }}{\left({\mathrm{Image}}_{1}^{j}\left[i\right]-{\mathrm{Image}}_{2}^{j}\left[i\right]\right)}^{2}$ is the mean square error at axis j (with size $N_{j}$) of images described as tensors, ${\mathrm{Image}}_{Original}$ is the original image, ${\mathrm{Image}}_{Coded}$ is the image produced by the trained ANN mimicking the image after HEVC compression, ${\mathrm{Image}}_{Hevc}$ is the image obtained after HEVC compression, CRF (constant-rate-factor) is the coefficient determining the degree of HEVC compression, $MSE_{OrgCodImg}$ is the mean square error of the image produced by the coder relative to the original image, $MSE_{HevcCodImg}$ is the mean square error of the image produced by the DNN coder in relation to the image obtained after HEVC compression, i is the training epoch number, $\mathrm{EpochChange}\left(i\right)$ is the modification value of the error function calculated on the basis of the epoch number, $L_{CodLoss}\left(i\right)$ is the encoder error, $Var_{\mathrm{Cod}}\left(i\right)$ is the tensor of the encoder model weights, $L_{Cod}\left(i\right)$ is the gradient of the coder objective function.

The training process used the Adam Optimizer algorithm [53]. The optimal number to achieve convergence for DNN was to run 300 epochs of the training algorithm. For the first 200 epochs of training, the learning rate had a fixed value of 10^−4^, and for the next 100 epochs the coefficient was changed to 10^−5^, as it was found during the experiments that a reduction in the rate after the 200th epoch yielded the best training results.

The training set consisted of 4000 images randomly downloaded from the Internet, which were changed to a 128 × 128 resolution compatible with the input size of the coder and the other ANNs discussed further. The images, when loaded for training, were converted to the YUV420p color model, which means that from the standard RGB color model, which has dimensions of 128 × 128 × 3 (where the first two numbers indicate height and width and the last digit indicates the number of channels), the images were converted accordingly with the YUV420p color model to a size of 192 × 128 × 1, which is the image size at the input and output of the network.

The test set consisted of video frames that had been converted to the H.265 codec format with a compression coefficient of CRF=7. The testing process compared the individual frames read from the video and then calculated $MSE_{HevcCodImg}$.

The images ${\mathrm{Image}}_{Hevc}\left(CRF\right)$, being images after passing through the HEVC compression channel, were coded with a coefficient of CRF=24. The operation of the HEVC channel was implemented by sending ${\mathrm{Image}}_{Original}$ to the HEVC codec, which converted the video to H.265 format and saved it to a single-frame video file. To speed up the process of writing and reading encoded frames in the DNN training process, writing files to RAM via RamDisk was used.

The structure of all ANNs occurring in this manuscript is unified, which means that the encoder has the same structure as the decoder. The only deviation from this statement is the encoder structure, which takes as its input two arguments in the form of images that are respectively the carrier and the watermark, in the remaining part, the encoder structure is the same. Table 2, Table 3 and Table 4 show the DNN parameters of the encoder and decoder, as well as the coder.

With reference to the previous article, it should be emphasized that the Basic Layer, the model of which is shown in Table 2, has remained unchanged, while the structures of the preliminary network (Table 3) and of the encoder and decoder (Table 4) have been altered through downsizing and unification.

The network structure consists of the following layers: Convolution 2D, Concatenate and Batch Normalization. For convolutional layers, the LeakyReLU activation function having a negative slope coefficient of 10^−2^ is used.

All the system elements that are DNNs, together with the coder that is the training element, have a preliminary network that prepares the image for the input of the actual system element before the actual calculations are made. In each system component, the preliminary network has the same structure but is dedicated to image preparation for a specific system component. At this point, it is worth emphasizing that in the encoder structure, the preliminary network is present only for the watermarked image, while the image being the watermark is fed without preprocessing through the preliminary network directly to the encoder input.

Parameters relating to the coder are shown in Table 5.

### 3.3. Watermarking Learning Process

The process of training watermark embedding by the encoder and its extraction by the decoder is implemented through the use of an error feedback mechanism consisting of a reciprocal effect on the training process of decoder-to-encoder and encoder-to-decoder, similar to what happens when training GAN type DNNs. A more detailed description of the components of the training process follows from the objective function used:

(6) $$MSE_{EncCoder}=\mathrm{MSE}\left({\mathrm{Image}}_{OriginalHevc},{\mathrm{Image}}_{EncodedCoder}\right)$$

(7) $$MSE_{EncHevc}=\mathrm{MSE}\left({\mathrm{Image}}_{OriginalHevc},{\mathrm{Image}}_{EncodedHevc}\right)$$

(8) $$MSE_{EncOriginal}=\mathrm{MSE}\left({\mathrm{Image}}_{Original},{\mathrm{Image}}_{Encoded}\right)$$

(9) $$MSE_{DecCoder}=\mathrm{MSE}\left({\mathrm{Image}}_{ToHideHevc},{\mathrm{Image}}_{DecodedCoder}\right)$$

(10) $$MSE_{DecHevc}=\mathrm{MSE}\left({\mathrm{Image}}_{ToHideHevc},{\mathrm{Image}}_{DecodedHevc}\right)$$

(11) $$MSE_{DecOriginal}=\mathrm{MSE}\left({\mathrm{Image}}_{ToHide},{\mathrm{Image}}_{Decoded}\right)$$

(12) $$\mathrm{EpochChange}\left(i\right)=i\cdot 0.001$$

(13) $$L_{EncLoss}\left(i\right)=\left(\left(\left(1.01-\mathrm{EpochChange}\left(i\right)\right)\cdot \;MSE_{EncOriginal}\right)\;\;+\left(\left(0.50+\mathrm{EpochChange}\left(i\right)\right)\cdot MSE_{EncHevc}\right)\;\;+\left(0.50\cdot MSE_{EncCoder}\right)\right)\;i\;\epsilon \left(0;250\right\rangle$$

(14) $$L_{EncLoss}\left(i\right)=\left(\left(\left(1.01-\mathrm{EpochChange}\left(i\right)\right)\cdot MSE_{EncOriginal}\right)\;\;+\left(\left(0.50+\mathrm{EpochChange}\left(i\right)\right)\cdot MSE_{EncHevc}\right)\;\;+\left(0.50\cdot MSE_{EncCoder}\right)\right)\;i\;\epsilon \left(251;500\right\rangle$$

(15) $$L_{DecLoss}\left(i\right)=\left(\left(\left(0.75\cdot \mathrm{EpochChange}\left(i\right)\right)\cdot MSE_{DecOriginal}\right)\;\;+\left(\left(0.50+\mathrm{EpochChange}\left(i\right)\right)\cdot MSE_{DecHevc}\right)\;\;+\left(0.50\cdot MSE_{DecCoder}\right)\right)\;i\;\epsilon \left(0;250\right\rangle$$

(16) $$L_{DecLoss}\left(i\right)=\left(\left(\left(0.75\cdot \mathrm{EpochChange}\left(i\right)\right)\cdot MSE_{DecOriginal}\right)\;\;+\left(\left(0.50+\mathrm{EpochChange}\left(i\right)\right)\cdot MSE_{DecHevc}\right)\;\;+\left(0.50\cdot MSE_{DecCoder}\right)\right)\;i\;\epsilon \left(251;500\right\rangle$$

(17) $$L_{EncFinalLoss}\left(i\right)=\left(L_{EncLoss}\left(i\right)+\left(0.56\cdot L_{DecLoss}\left(i\right)\right)\right)$$

(18) $$L_{DecFinalLoss}\left(i\right)=\left(L_{DecLoss}\left(i\right)+\left(0.56\cdot L_{EncLoss}\left(i\right)\right)\right)$$

(19) $$\nabla L_{Enc}\left(i\right)=\frac{\partial L_{EncFinalLoss}\left(i\right)}{\partial Var_{\mathrm{Enc}}\left(i\right)}$$

(20) $$\nabla L_{Dec}\left(i\right)=\frac{\partial L_{DecFinalLoss}\left(i\right)}{\partial Var_{\mathrm{Dec}}\left(i\right)}$$

where ${\mathrm{Image}}_{OriginalHevc}$ is the original image after HEVC channel compression, ${\mathrm{Image}}_{Encoded}$ is the image with an embedded watermark, ${\mathrm{Image}}_{EncodedHevc}$ is the image with an embedded watermark after HEVC channel compression, ${\mathrm{Image}}_{EncodedCoder}$ is the image with an embedded watermark after coder DNN compression, ${\mathrm{Image}}_{ToHide}$ is the hidden image constituting a watermark, ${\mathrm{Image}}_{ToHideHevc}$ is the hidden image constituting a watermark after HEVC channel compression, ${\mathrm{Image}}_{Decoded}$ is the image recovered with a hidden watermark, ${\mathrm{Image}}_{DecodedHevc}$ is the image recovered with a hidden watermark after HEVC channel compression, ${\mathrm{Image}}_{DecodedCoder}$ is the image recovered with the hidden watermark after compression by the coder DNN, $MSE_{EncCoder}$ is the mean square error of the image with the embedded watermark after compression of the HEVC channel, and the image with the embedded watermark after compression by the coder DNN, $MSE_{EncHevc}$ is the mean square error of the original image after compression of the HEVC channel, and the image with the embedded watermark after compression of the HEVC channel, $MSE_{EncOriginal}$ is the mean square error of the original image, and the image with the embedded watermark, $MSE_{DecCoder}$ is the mean square error of the image with the hidden watermark after compression of the HEVC channel, and the image recovered with the hidden watermark after compression by the coder, $MSE_{DecHevc}$ is the mean square error of the image with a hidden watermark after compression of the HEVC channel, and the image recovered with the hidden watermark after compression of the HEVC channel, $MSE_{DecOriginal}$ is the mean squared error of the image with a hidden watermark, and the image recovered with the hidden watermark, i is the training epoch number, $\mathrm{EpochChange}\left(i\right)$ is the value modifying the error function calculated in dependence of epoch number, $L_{EncLoss}\left(i\right)$ is the encoding error (embedding the watermark while maintaining transparency), $L_{DecLoss}\left(i\right)$ is the decoding error (of recovering the watermark), $L_{EncFinalLoss}\left(i\right)$ is the proportionally summed encoder error including the decoder error, $L_{DecFinalLoss}\left(i\right)$ is the proportionally summed encoder error taking into account the encoder error, $Var_{\mathrm{Enc}}\left(i\right)$ is the tensor representing the weights of the encoder model, $Var_{\mathrm{Dec}}\left(i\right)$ is the tensor representing the weights of the decoder model, $\nabla L_{Enc}\left(i\right)$ is the gradient of the encoder error function, $\nabla L_{Dec}\left(i\right)$ is the gradient of the decoder error function.

For the purposes of the training process, the same training set and test set were adopted as for the DNN coder, with the difference that on successive frames from the video constituting the test set $MSE_{EncHevc}$ and $MSE_{DecHevc}$ were calculated. Watermarks ${\mathrm{Image}}_{\mathrm{ToHide}}$ were generated by ASPA from randomly generating binary strings. For the HEVC compression channel, the same coefficient value as in the coder DNN training process was assumed equal to CRF=24. The training process also used the Adam Optimizer training method with a fixed learning rate of 10^−4^.

Parameters relating to the encoder and decoder are shown in Table 6.

### 3.4. Edge Effect

**Figure 3:**
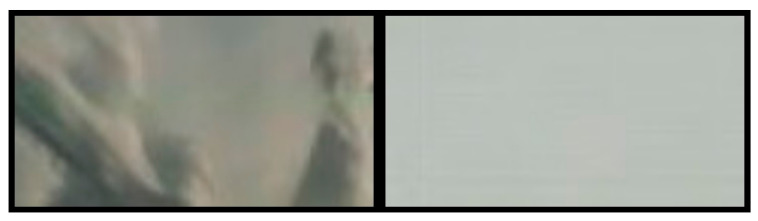
Preview of the edge effect: Left for a poorly trained network, right for a better trained network.

When embedding a watermark in an image larger than the 128 × 128 resolution of the encoder input image, it is necessary to divide the input image into fragments that match the encoder input. During the watermark embedding process, an edge effect may occur (Figure 3), consisting of a perimeter marking of the encoded image section, which is noticeable upon zooming in.

This effect can be present with a well-trained ANN (correctly working over most of the image area with a negligible edge effect up to a few pixels in width) and can be transparent and therefore not present in practice for a correctly trained ANN that has a very high level of image reproduction relative to the original image as in the case of the network trained for the purposes of the research presented in this article.

The edge effect can be easily eliminated by re-encoding the parts of the image where it is visible. This increases the time needed to embed the watermark, however, it ensures high image quality without any visible distortion in the form of an edge effect.

## 4. Results

This section will present the results of the studies successively for: The DNN coder mimicking HEVC compression, the encoder, and the decoder. The results of the studies are presented graphically in the form of graphs and video frames preview and alongside tables consisting of the results of the individual experiments. The research was conducted on a Linux operating system using the TensorFlow version 2.9.1 machine learning library and the FFmpeg version 4.4.2 video handling library. The source codes were written in Python version 3.10.4.

### 4.1. HEVC Compression Research Results

**Figure 4:**
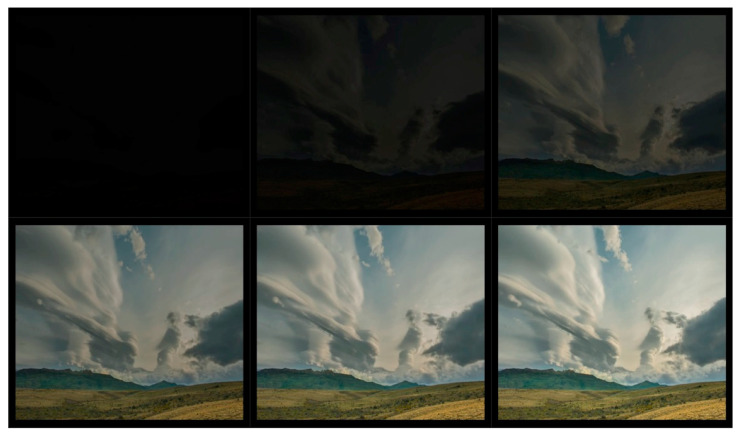
A preview of the frames from the test video used for the research presentation is subsequently (from the top left): 1, 5, 10, 20, 25, and 30.

**Figure 5:**
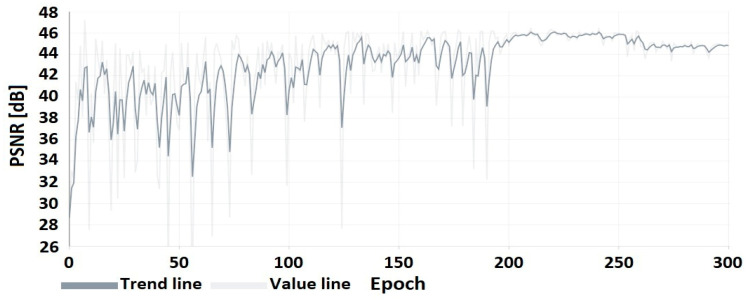
PSNR of the coder image (trained on CRF = 24) to the image after HEVC compression.

**Figure 6:**
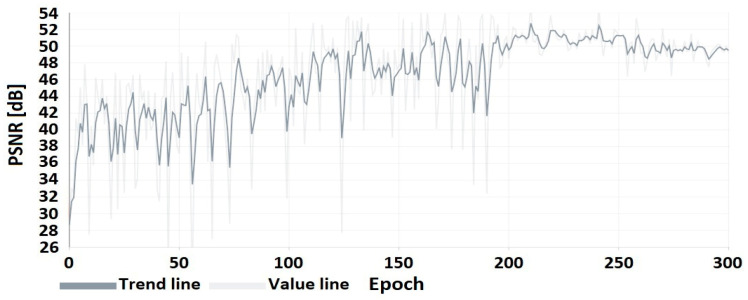
PSNR of the coder image (trained on CRF = 24) to the original image.

The results of the DNN coder mimicking HEVC coding are presented as a comparison of the image obtained at the output of the DNN coder to the image obtained at the output of the HEVC codec (Table 7) for selected CRF values from the full compression range ⟨0;51⟩. A preview of the frames from the test video used for the research presentation is shown in Figure 4. The flow of the coder training process is shown in Figure 5 and Figure 6.

The test set is made up of the individual initial frames of the test video, which are dimmed at the start and gradually brightened while movement occurs in the image [54]. This choice of a test set ensures that the proposed method is tested under more demanding conditions, as the compression coefficient for more dynamic motion scenes may increase, while for more static scenes, it may be reduced when using CRF. Unlike the QP (quantization parameter), which sets a fixed compression value for the entire video, the CRF allows the compression to be changed to a higher or lower value. In practical application, it is popularized to determine the degree of video compression using the CRF, so the results presented in this article will be using this parameter.

An additional factor influencing the selection of the test video is the presence of darkened and completely dark frames in the video, which is important as potential graphic artifacts can easily be seen in dark background. Because it is easier to hide a watermark (another image in the image) in a brightened image, there is a tendency to brighten images. In order to test the encoder’s performance in this aspect as well, it was decided to also use darkened video frames in the test sample.

Analyzing the results in the table above, it is noticeable that the DNN coder tends to reproduce the darkened images more accurately. In the range of values ⟨0;16⟩ for CRF, the quality of the PNSR reproduction is above 50 dB, which is due to the fact that in this range of values, the compression is not expansive, which is further confirmed in the column with the results of the comparison with the original uncompressed image.

One of the factors affecting the accuracy of the DNN coder’s representation of an image relative to an image encoded by H.265 is not only to consider the similarity of the coder’s output image to the HEVC codec’s output image but also to consider the similarity to the original image. For the CRF value with which the coder was trained, the PSNR value oscillates closer to the order of 50 dB than 40 dB, as is the case for compression coefficient values above 30.

The graphs from the training process up to epoch 200 show an upward trend in the PSNR coefficient. In subsequent epochs, there is a reduction in oscillations resulting from a reduction in the learning factor, as described in the section on coder training.

### 4.2. Watermarking Research Results

**Figure 7:**
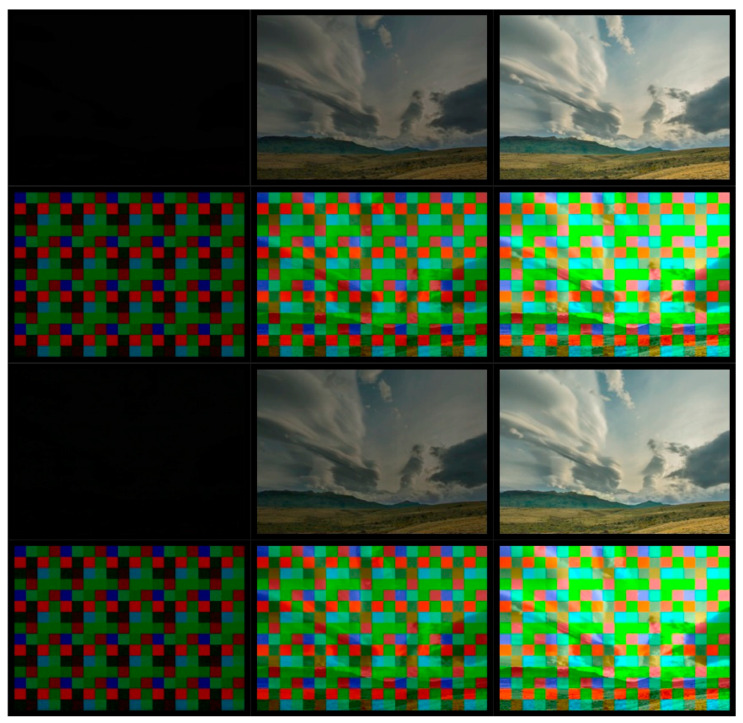
Preview of the results of watermark embedding and extraction under CRF = 20 compression conditions for: 1, 15, and 30 frame of test video for a 480 × 640 video fragment encoded with a 128 × 128 encoder (viewed from the left, the columns represent consecutive video frames, viewed from the top, the consecutive rows represent the original image, the watermark image, the watermarked image, and the recovered watermark).

**Figure 8:**
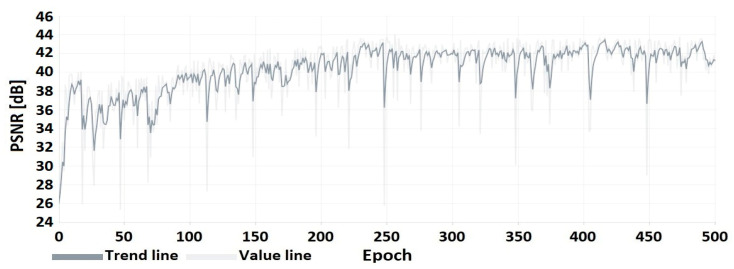
PSNR of the embedded watermarked image relative to the original image.

**Figure 9:**
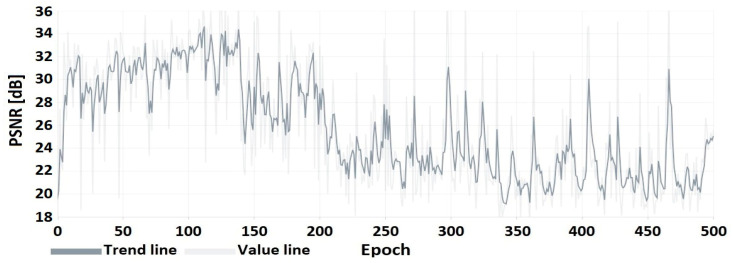
PSNR of the hidden image (watermark in the form of image) relative to the recovered image.

**Figure 10:**
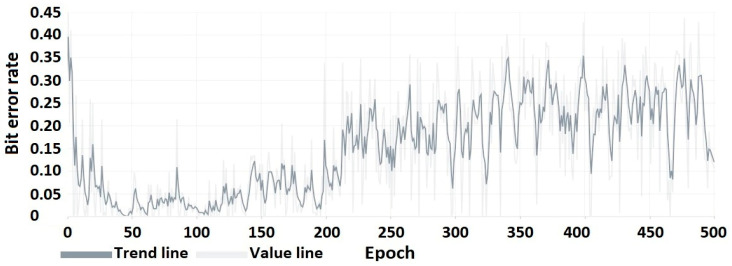
BER of the hidden message (watermark in the form of binary string).

The results of the proposed watermarking method will be discussed in this subsection. Firstly, the impact of watermarking will be discussed in terms of the accuracy of the reproduction of the watermarked carrier to its original form (Table 8). Next, the importance of changing the compression value and image resolution in the context of watermark survival will be discussed (Table 9, Table 10, Table 11 and Figure 7). Characteristics showing the training process are included at the end (Figure 8, Figure 9 and Figure 10).

Comparing the results in Table 7 and Table 8, there is an obvious reduction in image accuracy for the different HEVC compression values related to changes in the image created during the embedding process of the watermark. The change in carrier reproduction quality is more pronounced for low compression values and decreases as compression increases. This is due to the fact that DNN encoder was trained to embed the watermark under higher compression conditions.

Besides calculating MSE and PSNR coefficients, the quality of embedded video image frames in comparison to original frames was additionally assessed visually by an independent test group. The objective of the test was to compare embedded images (video frames) with their original counterpart. The tested person did not know which image was embedded and which was original. Every tested person was given 7 pairs of images. The objective of the tested person was to point out which image is original or asses pair as the same images. The formula to calculate the quality of the algorithm is given below:

(21) $$Q=\frac{L}{N}\cdot 100\%$$

where Q is the subjective assessment of the quality of the encoded image, L is the number of pairs of pictures identified as being the same or misclassified, N is the number of image pairs.

The obtained average result on the test group consisting of 15 tested persons was Q=62%. The obtained average result should be interpreted as a high similarity of the image with the embedded watermark to the original image, taking into account the results described below.

During the research, it turned out that in the test group, there were also people who were able to find subtle subjective differences after zooming in on the image, but were unable to indicate which image was original and which was modified, which meets the assumptions of the method, because the watermark is invisible to the human eye (the test person, who noticed the differences after zooming in on the image fragments, were not able to indicate which image from the pair was modified). This result proves the effectiveness of the method in practical application and is supported by the objective results obtained mathematically in the form of the values of the MSE and PSNR coefficients.

The change in compression coefficient expressed as CRF has a significant impact on the survival of the watermark under HEVC coding conditions. The changes in the image caused by the lower compression are not very invasive, which manifests itself in zero BER values. Based on the results in Table 9, it is noticeable that there is a tendency to correctly recover the watermark from all frames of the video test footage for a range of CRF values of ⟨0;22⟩. It is also possible to completely recover the watermark from individual video frames for CRF values not exceeding 24, however, it is more probable that for decoded watermark, BER would not be 0.

The previous publication [22] presented only the most efficient way of extracting values from the subsquares in the form of the median used in the ASPA operation. Watermark extraction results using the mean and the most frequent element (value) are also included in the results discussed. By placing the results of the quality of the decoded watermark in the form of a BER using these methods, the following conclusions can be drawn, as presented in the next paragraph.

The increase in compression, the invasiveness which causes increasingly significant changes, is well illustrated by the BER for information extraction using the most frequent element. In a 32 × 32 subsquare according to ASPA, the same values are embedded for each pixel. As the pixel values in the subsquare change due to compression, the number of pixels representing the embedded value is reduced. It is for this reason that, despite the appearance of non-zero BER values for the most frequently occurring element, recovery of the embedded value by the mean and median is possible. The median compared to the mean is a better way of extracting information, as it uses the mean results in larger BER values. This is due to the principle of the median, whose operation, unlike that of the mean, is not affected by changes in the extreme values in the set.

Table 10 shows the results of a recovered watermark in the context of a correctly recovered number of characters that correspond to the results obtained in Table 9.

The coded watermark by ASPA was a 16-character message with the following text „*WATERMARK_WAT22”.

Algorithm performance results when changing encoded image resolutions are shown in Table 11.

Changing the resolution of the encoded image significantly affects the quality of the recovered watermark. When the image resolution is changed in the form of a multiple reduction or increase of the encoded image, the watermark is kept according to the results in Table 11. However, if the image resolution reduces the subsquares for ASPA interpretation to 16 × 16, the watermark will degrade. This is due to the properties of the HEVC codec, which performs operations on subsquares in this resolution, which causes significant changes in the values for individual pixels and directly translates into the loss of the possibility of correct recovery of the watermark.

A possible solution to improve the possibility of complete recovery of the watermark is the use of error-correcting codes (ECC). ECC involve the addition of sufficient redundant data to the main information, therefore, the use of such a solution reduces the capacity of the watermark.

In the case of the proposed method using ASPA, the use of ECC methods like Turbocodes [55,56] or Polar codes [57,58] would significantly increase the potential recovery of the watermark even when BER values are not equal to 0. The acceptable value of BER (when BER > 0) for which the watermark could be retrieved will be depended on the used ECC method and the size of the correction code. However, it will reduce the capacity of the watermark because subsquares that could be used for main information will be occupied by error correction codes.

The introduction of ECC schemes for ASPA purposes will increase the probability of full watermark recovery even when BER > 0 after the decoding process with higher compression levels of HEVC. It is also worth underlining that adding a neural network to the learning process compression channel to retrieve embedded watermark creates own ECC mechanisms, which are based on learnt filters. By improving the architecture of DNN encoder and decoder, the probability of full watermark recovery will be increased.

A preview of the encoder and decoder results is shown in Figure 7.

The following characteristics (Figure 8, Figure 9 and Figure 10) show the encoder and decoder training process. These elements were trained on an autoencoder basis for 500 training epochs.

### 4.3. Comparison with Other Methods

In order to check the performance of the proposed method, a comparison was made with other state-of-the-art (SOTA) methods, which are listed in the literature review. In order to compare the methods, the HEVC compression value was assumed to be 16 and the resolution of the test video to be 416 × 240. The results of the comparison of watermark embedding methods under HEVC compression conditions are shown in Table 12.

The methods presented in Table 12 represent different approaches to embedding a watermark in video under HEVC compression conditions. In the first method, the watermark is embedded as intra-prediction residual pixels of 4 × 4 luminance transform blocks in the spatial domain [16]. In the second method compared, the prediction modes of selected 4 × 4 intra-prediction blocks are changed [17]. In the next method, the watermark is embedded as the multi-coefficients of the selected 4 × 4 luminance discrete sine transform blocks [51].

The previously proposed method differs from the one presented in this article in efficiency. The watermark capacity has been increased along with its resistance to HEVC compression while increasing the PSNR value. The architecture of the DNNs used, and the way they are trained has been changed, however, the way the watermark is embedded and restored has remained based on the operation of the ASPA.

The average PSNR for the proposed method is dependent on the compression coefficient, however, it is greater than 44 dB in the HEVC compression range, allowing it to be recovered. The PSNR for the proposed method has a higher value than for the previously proposed method and is close to the method having the highest value of the compared SOTA methods.

The calculation time for the proposed method is noticeably different from the other methods compared. The reason for this state is the non-optimized structure of the ANN for real-time processing. However, the lack of optimization of the ANN structure is not the main reason for the long calculation time, it is caused by the suboptimal division of the image into fragments corresponding to the resolution matching the ANN input. The main factor that extends the watermark embedding process is the support for the YUV420p color model and the associated image storage.

An optimization involving the parallelization of the processes of obtaining and overwriting image fragments would significantly speed up computation. The time calculated for the implementation that does not include the parallelized calculations for watermark embedding is 81.13 s. The calculation time for the sequential calculation on previously prepared image fragments of the YUV420p color model with a resolution corresponding to the size of the encoder input is 0.74 s. By paralleling the calculations performed by the ANN, this time can theoretically be reduced below 0.1 s.

The resolution of the test image to unify the comparison was 416 × 240, for which the compared methods have a watermark capacity of 100 bits. The encoder of the proposed method operates natively at a resolution of 128 × 128, so it needs to perform eight encode operations to embed the watermark. The native image resolution at which the encoder operates, which is 128 × 128, embeds 96 watermark bits. For comparison purposes, the same watermark was embedded over the entire area of the 416 × 240 video frames. However, other bit strings can be encoded in each of the 128 × 128 image areas, which together will make up the larger watermark. For example, by embedding a watermark on a similar resolution of 256 × 256, a watermark of 384 bits can be embedded. The proposed method has a higher capacity than the compared methods and has the potential to increase it further.

## 5. Conclusions

Embedding a watermark in a video, taking into account maximizing the accuracy of the watermarked image in relation to the original image under the compression conditions introduced by the video codec, is a complex issue. For solutions using DNNs, it is worth considering the required hardware resources for DNN training and their subsequent use. The solution presented in this publication allows the DNN to operate natively for a 128 × 128 image to be freely scaled to larger images through the use of a sliding window, which also allows for a potential increase in watermark capacity within a single video frame.

Compression introduced by HEVC above a CRF of 16 is more challenging for the encoder and decoder training process for the purposes of watermark embedding and recovery. This state of affairs is due to the complex HEVC compression characteristics. Appropriate implementation of the training process by introducing an additional training element, the DNN coder, which mimics the HEVC compression channel, accelerates convergence at the training of HEVC compression characteristics, which enables the encoder and decoder to operate at higher compression values.

The presented method takes into account not only HEVC compression but also the image compression introduced through the application of YUV420p. The use of the YUV color model in the YUV420p variant further affects the compression of the image and therefore reduces the size of the output video file, however, it is also an additional issue to consider when embedding the watermark. The issue presented in the previous sentence is solved through ASPA, which allows the size of the image fragment used to store the watermark bit string to be selected. By applying synergies in the application of ASPA and HEVC compression together with YUV420p in the form of the appropriate use of the subsquares present in these components of the method under discussion, it is possible to correctly embed and recover the watermark despite a fourfold reduction in capacity for chrominance through the application of YUV420p.

The analysis of the results of the conducted tests of the proposed method confirms the possibility of completely recovering the watermark from a single frame in terms of changes in the value ⟨0;22⟩ of the compression coefficient determined by the CRF. It is also possible to completely recover the watermark from individual video frames for CRF values not exceeding 24, however, it is more probable that for decoded watermark, BER would not be 0.

Further research is needed into the possibility of more accurate reproduction of an image with embedded watermarked compared to the original. The issues of increasing the capacity of the watermark itself and increasing its resistance to changes in its carrier while reducing the time required for embedding would also require further exploration.

In order to improve the coefficients describing the accuracy of the reproduction of the embedded watermarked image to the original image, the error function used during the training process should be further modified alongside the performance improvement of mimicking the HEVC compression by the DNN coder, which is the training element of the H.265 codec characteristics for encoder and decoder. Further increases in watermark capacity can be achieved by modifying the ASPA parameters. The application of an additional element, in the form of a variable decoder extension dedicated to different types of interference, will improve the correctness of watermark recovery from a carrier subjected to various watermark distorting operations. The introduction of ECC schemes for ASPA purposes will increase the probability of full watermark recovery.

Another possibility to improve the performance of the decoder subsystem is to use the video frame sequence with a histogram calculation to map most of the repeated values between frames at the output of the DNN decoder, enhancing the watermark recovery. Moreover, creating a frame-pack-based system for watermark augmentation and enhancement via ECC will increase the overall system performance.

Further reduction of DNN structures and implementation of the optimization of the parallelization of the watermark embedding, and extraction algorithms will contribute to the reduction of the computational time.

## Figures and Tables

**Table 1:** Value ranges for encoding and decoding digits.

Digit	Value Range	RGB Value Range
0	⟨0;0.125⟩	⟨0;31.875⟩
1	(0.125;0.25⟩	(31.875;63.75⟩
2	(0.25;0.375⟩	(63.7;95.625⟩
3	(0.375;0.5⟩	(95.625;127.5⟩
4	(0.5;0.625⟩	(127.5;159.375⟩
5	(0.625;0.75⟩	(159.375;191.25⟩
6	(0.75;0.875⟩	(191.25;223.125⟩
7	(0.875;1⟩	(223.125;255⟩

**Table 2:** Basic Layer (BL) processing model.

Layer Number	Layer Type
1	Convolution 2D layer
2	Batch Normalization
3	Convolution 2D layer
4	Batch Normalization
5	Convolution 2D layer
6	Batch Normalization

**Table 3:** Preliminary, convolutional neural network model.

Layer Number	Layer Type	Parameters
1	BL	activation function: LeakyReLU
		kernel size: 3 × 3
		filter number: 63
2	BL	activation function: LeakyReLU
		kernel size: 5 × 5
		filter number: 63
3	Concatenate ((1, 2), axis = 3)	-
4	BL	activation function: LeakyReLU
		kernel size: 5 × 5
		filter number: 63
5	BL	activation function: LeakyReLU
		kernel size: 3 × 3
		filter number: 63
6	Concatenate ((4, 5), axis = 3)	-

**Table 4:** A model of the convolutional neural network: Coding, encoding, and decoding.

Layer Number	Layer Type	Parameters
1	BL	activation function: LeakyReLU
		kernel size: 3 × 3
		filter number: 63
2	BL	activation function: LeakyReLU
		kernel size: 5 × 5
		filter number: 63
3	BL	activation function: LeakyReLU
		kernel size: 7 × 7
		filter number: 63
4	Concatenate ((1, 2, 3), axis = 3)	-
5	Batch Normalization (4)	
6	BL	activation function: LeakyReLU
		kernel size: 3 × 3
		filter number: 63
7	BL	activation function: LeakyReLU
		kernel size: 7 × 7
		filter number: 63
8	Concatenate ((6, 7), axis = 3)	-
9	Batch Normalization (8)	
10	BL	activation function: LeakyReLU
		kernel size: 7 × 7
		filter number: 63
11	BL	activation function: LeakyReLU
		kernel size: 5 × 5
		filter number: 63
12	BL	activation function: LeakyReLU
		kernel size: 3 × 3
		filter number: 63
13	Concatenate ((10, 11, 12), axis = 3)	-
	Batch Normalization (13)	
14	Convolution 2D layer	activation function: LeakyReLU
		kernel size: 1

**Table 5:** Parameters of the coder.

Type	InputTensor Size	Number of Weights	Size on Disk	Processing Time for a Single Tensor [ms]	GPU Processor
Coder	(192, 128, 1)	5771494	69.00 MB	61.551	GeForce 1080Ti GTX 11GB

**Table 6:** Parameters of the encoder and decoder.

Type	Input Tensor Size	Number of Weights	Size on Disk	Processing Time for a Single Tensor [ms]	GPU Processor
Encoder	(2, 192, 128, 1)	5776723	69.10 MB	62.234	GeForce 1080Ti GTX 11GB
Decoder	(192, 128, 1)	5771494	69.00 MB	61.424	

**Table 7:** Encoder operation results for a 1920 × 1080 video fragment compressed with a 128 × 128 encoder trained for CRF = 24 compared to the original image and to a video fragment compressed by HEVC compression for MSE and PSNR changes in CRF.

Frame #	Original	CRF 0	CRF 7	CRF 16	CRF 23	CRF 24	CRF 28	CRF 31	CRF 41	CRF 51
1	PSNR ^1^: 52.18	PSNR: 52.18	PSNR: 52.18	PSNR: 52.17	PSNR: 52.10	PSNR: 52.08	PSNR: 51.97	PSNR: 51.78	PSNR: 50.82	PSNR: 44.27
	MSE ^2^: 4.29 × 10^−7^	MSE: 4.36 × 10^−7^	MSE: 4.36 × 10^−7^	MSE: 4.77 × 10^−7^	MSE: 6.33 × 10^−7^	MSE: 6.69 × 10^−7^	MSE: 8.83 × 10^−7^	MSE: 1.19 × 10^−6^	MSE: 2.70 × 10^−6^	MSE: 1.95 × 10^−5^
5	PSNR: 51.73	PSNR: 51.72	PSNR: 51.72	PSNR: 51.51	PSNR: 51.03	PSNR: 50.94	PSNR: 50.35	PSNR: 49.72	PSNR: 47.11	PSNR: 44.32
	MSE: 5.00 × 10^−7^	MSE: 5.23 × 10^−7^	MSE: 5.23 × 10^−7^	MSE: 1.04 × 10^−6^	MSE: 2.27 × 10^−6^	MSE: 2.51 × 10^−6^	MSE: 4.07 × 10^−6^	MSE: 5.58 × 10^−6^	MSE: 1.46 × 10^−5^	MSE: 3.40 × 10^−5^
10	PSNR: 53.44	PSNR: 53.42	PSNR: 53.42	PSNR: 52.75	PSNR: 51.38	PSNR: 51.11	PSNR: 49.65	PSNR: 48.44	PSNR: 44.25	PSNR: 40.68
	MSE: 6.42 × 10^−7^	MSE: 6.71 × 10^−7^	MSE: 6.70 × 10^−7^	MSE: 1.57 × 10^−6^	MSE: 3.93 × 10^−6^	MSE: 4.52 × 10^−6^	MSE: 8.13 × 10^−6^	MSE: 1.18 × 10^−5^	MSE: 3.56 × 10^−5^	MSE: 8.35 × 10^−5^
15	PSNR: 52.33	PSNR: 52.31	PSNR: 52.31	PSNR: 51.60	PSNR: 50.19	PSNR: 49.89	PSNR: 48.37	PSNR: 47.02	PSNR: 42.27	PSNR: 38.52
	MSE: 7.63 × 10^−7^	MSE: 7.97 × 10^−7^	MSE: 7.97 × 10^−7^	MSE: 2.03 × 10^−6^	MSE: 5.07 × 10^−6^	MSE: 5.85 × 10^−6^	MSE: 1.06 × 10^−5^	MSE: 1.61 × 10^−5^	MSE: 5.64 × 10^−5^	MSE: 1.38 × 10^−4^
20	PSNR: 52.54	PSNR: 52.52	PSNR: 52.52	PSNR: 51.66	PSNR: 49.98	PSNR: 49.63	PSNR: 47.81	PSNR: 46.22	PSNR: 40.82	PSNR: 36.69
	MSE: 8.48 × 10^−7^	MSE: 8.82 × 10^−7^	MSE: 8.83 × 10^−7^	MSE: 2.38 × 10^−6^	MSE: 6.13 × 10^−6^	MSE: 7.10 × 10^−6^	MSE: 1.34 × 10^−5^	MSE: 2.09 × 10^−5^	MSE: 8.07 × 10^−5^	MSE: 2.12 × 10^−4^
25	PSNR: 51.92	PSNR: 51.91	PSNR: 51.91	PSNR: 51.04	PSNR: 49.37	PSNR: 49.01	PSNR: 47.17	PSNR: 45.49	PSNR: 39.78	PSNR: 35.34
	MSE: 1.02 × 10^−6^	MSE: 1.06 × 10^−6^	MSE: 1.06 × 10^−6^	MSE: 2.86 × 10^−6^	MSE: 7.12 × 10^−6^	MSE: 8.22 × 10^−6^	MSE: 1.53 × 10^−5^	MSE: 2.46 × 10^−5^	MSE: 1.03 × 10^−4^	MSE: 2.90 × 10^−4^
30	PSNR: 52.02	PSNR: 52.00	PSNR: 51.99	PSNR: 51.00	PSNR: 49.13	PSNR: 48.73	PSNR: 46.79	PSNR: 45.03	PSNR: 39.08	PSNR: 34.51
	MSE: 1.26 × 10^−6^	MSE: 1.30 × 10^−6^	MSE: 1.30 × 10^−6^	MSE: 3.34 × 10^−6^	MSE: 8.18 × 10^−6^	MSE: 9.45 × 10^−6^	MSE: 1.75 × 10^−5^	MSE: 2.81 × 10^−5^	MSE: 1.21 × 10^−4^	MSE: 3.52 × 10^−4^

^1^ Peak signal-to-noise ratio, ^2^ Mean squared error normalized over a range of values ⟨0;1⟩.

**Table 8:** Algorithm performance results for a 480 × 640 video fragment encoded with a 128 × 128 encoder trained for CRF = 24 for changes in CRF coefficient compared to a video fragment compressed by HEVC compression in the form of MSE and PSNR.

Frame #	CRF 0	CRF 7	CRF 12	CRF 16	CRF 20	CRF 22	CRF 23	CRF 24	CRF 25
1	PSNR ^1^: 49.61	PSNR: 49.61	PSNR: 49.57	PSNR: 49.61	PSNR: 49.98	PSNR: 50.22	PSNR: 50.40	PSNR: 50.57	PSNR: 51.01
	MSE ^2^: 1.09 × 10^−5^	MSE: 1.10 × 10^−5^	MSE: 1.10 × 10^−5^	MSE: 1.09 × 10^−5^	MSE: 1.00 × 10^−5^	MSE: 9.52 × 10^−6^	MSE: 9.13 × 10^−6^	MSE: 8.76 × 10^−6^	MSE: 7.93 × 10^−6^
5	PSNR: 50.13	PSNR: 50.13	PSNR: 49.94	PSNR: 49.65	PSNR: 49.73	PSNR: 49.82	PSNR: 49.89	PSNR: 50.08	PSNR: 50.27
	MSE: 9.70 × 10^−6^	MSE: 9.70 × 10^−6^	MSE: 1.01 × 10^−5^	MSE: 1.08 × 10^−5^	MSE: 1.06 × 10^−5^	MSE: 1.04 × 10^−5^	MSE: 1.03 × 10^−5^	MSE: 9.82 × 10^−6^	MSE: 9.41 × 10^−6^
10	PSNR: 47.99	PSNR: 47.98	PSNR: 47.79	PSNR: 47.48	PSNR: 47.38	PSNR: 47.32	PSNR: 47.24	PSNR: 47.35	PSNR: 47.34
	MSE: 1.59 × 10^−5^	MSE: 1.59 × 10^−5^	MSE: 1.67 × 10^−5^	MSE: 1.79 × 10^−5^	MSE: 1.83 × 10^−5^	MSE: 1.86 × 10^−5^	MSE: 1.89 × 10^−5^	MSE: 1.84 × 10^−5^	MSE: 1.85 × 10^−5^
15	PSNR: 47.62	PSNR: 47.62	PSNR: 47.63	PSNR: 47.03	PSNR: 46.91	PSNR: 46.78	PSNR: 46.74	PSNR: 46.75	PSNR: 46.73
	MSE: 1.73 × 10^−5^	MSE: 1.73 × 10^−5^	MSE: 1.83 × 10^−5^	MSE: 1.98 × 10^−5^	MSE: 2.04 × 10^−5^	MSE: 2.10 × 10^−5^	MSE: 2.12 × 10^−5^	MSE: 2.11 × 10^−5^	MSE: 2.12 × 10^−5^
20	PSNR: 46.74	PSNR: 46.74	PSNR: 46.49	PSNR: 46.17	PSNR: 45.96	PSNR: 45.84	PSNR: 45.84	PSNR: 45.79	PSNR: 45.69
	MSE: 2.12 × 10^−5^	MSE: 2.12 × 10^−5^	MSE: 2.24 × 10^−5^	MSE: 2.42 × 10^−5^	MSE: 2.54 × 10^−5^	MSE: 2.61 × 10^−5^	MSE: 2.61 × 10^−5^	MSE: 2.63 × 10^−5^	MSE: 2.70 × 10^−5^
25	PSNR: 46.35	PSNR: 46.35	PSNR: 46.07	PSNR: 45.75	PSNR: 45.52	PSNR: 45.36	PSNR: 45.34	PSNR: 45.26	PSNR: 45.13
	MSE: 2.32 × 10^−5^	MSE: 2.32 × 10^−5^	MSE: 2.47 × 10^−5^	MSE: 2.66 × 10^−5^	MSE: 2.81 × 10^−5^	MSE: 2.91 × 10^−5^	MSE: 2.93 × 10^−5^	MSE: 2.98 × 10^−5^	MSE: 3.07 × 10^−5^
30	PSNR: 46.03	PSNR: 46.03	PSNR: 45.75	PSNR: 45.41	PSNR: 45.12	PSNR: 45.00	PSNR: 44.94	PSNR: 44.84	PSNR: 44.73
	MSE: 2.50 × 10^−5^	MSE: 2.50 × 10^−5^	MSE: 2.66 × 10^−5^	MSE: 2.88 × 10^−5^	MSE: 3.08 × 10^−5^	MSE: 3.17 × 10^−5^	MSE: 3.21 × 10^−5^	MSE: 3.28 × 10^−5^	MSE: 3.36 × 10^−5^

^1^ Peak signal-to-noise ratio, ^2^ Mean squared error normalized over a range of values ⟨0;1⟩.

**Table 9:** Algorithm performance results for a 480 × 640 video fragment encoded with a 128 × 128 encoder trained for CRF = 24 for changes in CRF coefficient in the form of a BER depending on the watermark reading variant.

Frame #	CRF 0	CRF 7	CRF 12	CRF 16	CRF 20	CRF 22	CRF 23	CRF 24	CRF 25
1	AVG ^1^: 0	AVG: 0	AVG: 0	AVG: 0	AVG: 0	AVG: 0	AVG: 0	AVG: 0.0536	AVG: 0.1518
	COM ^2^: 0	COM: 0	COM: 0	COM: 0	COM: 0	COM: 0.0804	COM: 0.0714	COM: 0.2143	COM: 0.3571
	MED ^3^: 0	MED: 0	MED: 0	MED: 0	MED: 0	MED: 0	MED: 0	MED: 0.0179	MED: 0.0625
5	AVG: 0	AVG: 0	AVG: 0	AVG: 0	AVG: 0	AVG: 0	AVG: 0.0089	AVG: 0.0089	AVG: 0.1071
	COM: 0	COM: 0	COM: 0	COM: 0	COM: 0.0357	COM: 0.0447	COM: 0.0893	COM: 0.25	COM: 0.2589
	MED: 0	MED: 0	MED: 0	MED: 0	MED: 0	MED: 0	MED: 0.0089	MED: 0.0089	MED: 0.0536
10	AVG: 0	AVG: 0	AVG: 0	AVG: 0	AVG: 0	AVG: 0.0089	AVG: 0.0089	AVG: 0.0089	AVG: 0.1161
	COM: 0	COM: 0	COM: 0	COM: 0	COM: 0.0179	COM: 0.0357	COM: 0.1429	COM: 0.2679	COM: 0.4821
	MED: 0	MED: 0	MED: 0	MED: 0	MED: 0	MED: 0	MED: 0	MED: 0	MED: 0.0446
15	AVG: 0	AVG: 0	AVG: 0	AVG: 0	AVG: 0	AVG: 0.0089	AVG: 0.0089	AVG: 0.0625	AVG: 0.1875
	COM: 0	COM: 0	COM: 0	COM: 0	COM: 0.0179	COM: 0.0804	COM: 0.2768	COM: 0.3482	COM: 0.4464
	MED: 0	MED: 0	MED: 0	MED: 0	MED: 0	MED: 0	MED: 0.0089	MED: 0.0089	MED: 0.0714
20	AVG: 0	AVG: 0	AVG: 0	AVG: 0	AVG: 0	AVG: 0.0089	AVG: 0.0089	AVG: 0.0714	AVG: 0.2143
	COM: 0	COM: 0	COM: 0	COM: 0	COM: 0.0714	COM: 0.1429	COM: 0.25	COM: 0.4375	COM: 0.5
	MED: 0	MED: 0	MED: 0	MED: 0	MED: 0	MED: 0	MED: 0.0089	MED: 0.0089	MED: 0.1071
25	AVG: 0	AVG: 0	AVG: 0	AVG: 0	AVG: 0	AVG: 0	AVG: 0.0179	AVG: 0.0357	AVG: 0.2321
	COM: 0	COM: 0	COM: 0	COM: 0	COM: 0.0179	COM: 0.1161	COM: 0.3839	COM: 0.3839	COM: 0.3571
	MED: 0	MED: 0	MED: 0	MED: 0	MED: 0	MED: 0	MED: 0.0089	MED: 0.0089	MED: 0.0893
30	AVG: 0	AVG: 0	AVG: 0	AVG: 0	AVG: 0	AVG: 0	AVG: 0.0179	AVG: 0.0714	AVG: 0.1607
	COM: 0	COM: 0	COM: 0	COM: 0.0179	COM: 0.0179	COM: 0.1339	COM: 0.0179	COM: 0.3839	COM: 0.4911
	MED: 0	MED: 0	MED: 0	MED: 0	MED: 0	MED: 0	MED: 0.0089	MED: 0.0179	MED: 0.0536

^1^ Bit error rate of the mean, ^2^ Bit error rate of the most frequently occurring element (value), ^3^ Bit error rate of the median.

**Table 10:** Algorithm performance results for a 480 × 640 video fragment encoded with a 128 × 128 encoder trained for CRF = 24 for changes in CRF coefficient in the form of a number of correctly decoded characters.

Frame #	CRF 0	CRF 7	CRF 12	CRF 16	CRF 20	CRF 22	CRF 23	CRF 24	CRF 25
1	CHAR ^1^: 16	CHAR: 16	CHAR: 16	CHAR: 16	CHAR: 16	CHAR: 16	CHAR: 16	CHAR: 15	CHAR: 13
5	CHAR: 16	CHAR: 16	CHAR: 16	CHAR: 16	CHAR: 16	CHAR: 16	CHAR: 15	CHAR: 15	CHAR: 14
10	CHAR: 16	CHAR: 16	CHAR: 16	CHAR: 16	CHAR: 16	CHAR: 16	CHAR: 16	CHAR: 16	CHAR: 14
15	CHAR: 16	CHAR: 16	CHAR: 16	CHAR: 16	CHAR: 16	CHAR: 16	CHAR: 15	CHAR: 15	CHAR: 12
20	CHAR: 16	CHAR: 16	CHAR: 16	CHAR: 16	CHAR: 16	CHAR: 16	CHAR: 15	CHAR: 15	CHAR: 12
25	CHAR: 16	CHAR: 16	CHAR: 16	CHAR: 16	CHAR: 16	CHAR: 16	CHAR: 15	CHAR: 15	CHAR: 13
30	CHAR: 16	CHAR: 16	CHAR: 16	CHAR: 16	CHAR: 16	CHAR: 16	CHAR: 15	CHAR: 14	CHAR: 13

^1^ Number of correctly decoded characters.

**Table 11:** Algorithm performance results for an encoded video fragment with a 128 × 128 encoder compressed with CRF = 16 when changing the video resolution: 96 bits decoding representation in the form of BER.

Frame #	From 512 × 512 to 128 × 128	From 512 × 512 to 256 × 256	From 512 × 512 to 480 × 640	From 512 × 512 to 1024 × 1024	From 768 × 512 to 384 × 256	From 1024 × 1024 to 256 × 256
1	BER ^1^: 0.531250	BER: 0	BER: 0.468750	BER: 0	BER: 0.510417	BER: 0.510417
5	BER: 0.479167	BER: 0	BER: 0.500000	BER: 0	BER: 0.489583	BER: 0.479167
10	BER: 0.395833	BER: 0	BER: 0.489583	BER: 0	BER: 0.437500	BER: 0.437500
15	BER: 0.427083	BER: 0	BER: 0.427083	BER: 0	BER: 0.468750	BER: 0.427083
20	BER: 0.437500	BER: 0	BER: 0.406250	BER: 0	BER: 0.468750	BER: 0.416667
25	BER: 0.447917	BER: 0	BER: 0.406250	BER: 0	BER: 0.416667	BER: 0.375000
30	BER: 0.416667	BER: 0	BER: 0.406250	BER: 0	BER: 0.427083	BER: 0.364583

^1^ Bit error rate of the median.

**Table 12:** Method comparison.

Type	Average PSNR (dB)	Capacity (Bits/Frame Size)	(Embedding Time/Extraction Time) (ms) for Test Frame with 416 × 240 Resolution	Hardware
**Method 1** **Zhou et al.** [16]	47.519	100 bits/416 × 240	32.478/5.622	3.30 GHz CPU, 4 GB RAM
**Method 2** **Gaj et al.** [17]	46.415	100 bits/416 × 240	36.855/5.048	3.30 GHz CPU, 4 GB RAM
**Method 3** **Liu et al.** [51]	45.462	100 bits/416 × 240	34.058/5.997	3.30 GHz CPU, 4 GB RAM
**Previously proposed method** [22]	42.617	80 bits/128 × 128	34,457.92/3759.72	Geforce 1080Ti GTX 11 GB, 32 GB RAM
**Proposed method**	47.299	96 bits/128 × 128	81,132.13 ^1^/82,223.74 ^1^ 735.101 ^2^/725.861 ^2^	Geforce 1080Ti GTX 11 GB, 32 GB RAM

^1^ Time calculated for an implementation that does not include parallelized calculations, ^2^ Time of calculations performed sequentially on beforehand prepared image fragments with a resolution corresponding to the size of the encoder and decoder inputs.

## Data Availability

Not applicable.
